# Associations between lifestyle behaviours and optimal wellbeing in a diverse sample of New Zealand adults

**DOI:** 10.1186/s12889-016-2755-0

**Published:** 2016-01-22

**Authors:** Kate B. Prendergast, Grant M. Schofield, Lisa M. Mackay

**Affiliations:** Auckland University of Technology, Private Bag 92006, Auckland, 1142 New Zealand

**Keywords:** Positive psychology, Population survey, Positive health, Physical activity, Nutrition, Sleep

## Abstract

**Background:**

In positive psychology optimal wellbeing is considered a broad, multi-dimensional construct encompassing both feelings and functioning. Yet, this notion of wellbeing has not been translated into public health. The purpose of this study is to integrate public health and positive psychology to determine associations between lifestyle behaviours and optimal wellbeing in a diverse sample of New Zealand adults.

**Methods:**

A web-based survey design was employed to collect data. Participants reported on their wellbeing and lifestyle behaviours including nutrition, exercise, sedentary behaviour, and sleep. Optimal wellbeing was calculated using a multi-dimensional scale designed to mirror the internationally recognised diagnostic criteria for mental disorders. Binary logistic regression was used to calculate associations between 10 lifestyle behaviours and optimal wellbeing.

**Results:**

Of the total sample (*n* = 9514), 24 % met the criteria for optimal wellbeing. Compared to reference groups, the association with optimal wellbeing was greater for those who reported exercising ≥7 times/week (odds ratio: 1.61, 95 % confidence interval: 1.22–2.13, *p* < 0.01) and sitting “almost none of the time” (1.87, 1.01–3.29, *p* < 0.01). Optimal wellbeing was lower for those reporting restless sleep “almost all of the time” (0.24, 95 % CI: 0.17–0.32 *p* < 0.01) and consuming sugary drinks 5–6 times/week (0.73, 95 % CI: 0.53–0.95, *p* < 0.05).

**Conclusions:**

Public health and positive psychology were integrated to provide support for a relationship between lifestyle behaviours and a multi-dimensional measure of optimal wellbeing. It is likely this relationship between lifestyle behaviours and optimal wellbeing is bidirectional giving rise to the debate that holistic approaches are needed to promote positive health.

## Background

In 1946 the World Health Organization defined health as “a state of complete physical, mental, and social well-being and not merely the absence of disease or infirmity” [1]. From this definition the notion of positive health, where health is considered beyond the absence of disease, emerged [2–4]. However, this concept of positive health remains somewhat elusive [2] as epidemiological work and public health guidelines continue to focus on preventing and restoring negative functioning rather than promoting positive health. In contrast to understanding pathology, far less is known about the behaviours and characteristics associated with positive health and optimal wellbeing.

Recognition that a fundamental shift was needed to study wellbeing in its own right has led to the emergence of the positive psychology field [5, 6]. Within positive psychology a broader, more complex notion of wellbeing has emerged [7–10]. Optimal wellbeing—or flourishing as it is also referred—is considered a multi-dimensional construct incorporating both hedonic (e.g. positive emotion, life satisfaction, and happiness) and eudaimonic (e.g. meaning and purpose, positive relationships, and engagement) aspects of wellbeing [5, 8, 10, 11]. There is now agreement that multi-dimensional measures of optimal wellbeing, which take into account hedonic and eudaimonic aspects of wellbeing, should be used to determine and characterise those individuals with the highest levels of wellbeing [5, 10].

Only recently have multi-dimensional measures of optimal wellbeing emerged which can be used to categorically determine those with the highest levels of wellbeing [5, 8]. In the largest wellbeing study to date, Huppert and So developed and tested a categorical measure of optimal wellbeing using a representative sample of 43,000 individuals from 22 European countries [5]. This measure of optimal wellbeing was developed using a conceptual framework specifically designed to mirror the internationally agreed methodology used to diagnose mental disorders [5]. Through a systematic examination of the symptoms of common mental disorders, generalised anxiety (ICD-10) and depression (DSM-IV), ten features representing optimal mental health were identified: happiness, vitality, optimism, resilience, self-esteem, emotional stability, engagement, meaning, competence, and positive relationships. Factor analysis, inter-item correlations, and data distribution indicated that optimal wellbeing required the presence of three factors: positive emotion (comprising happiness); positive characteristics (vitality, optimism, resilience, self-esteem, emotional stability); and positive functioning (engagement, meaning, competence, positive relationships). To be classified as having optimal wellbeing individuals are required to meet the criteria for positive emotion, four out of five features of positive characteristics, and three out of four features of positive functioning. This method of classifying the presence of optimal wellbeing is similar to that used to classify major depressive disorders where the presence of most, but not all, features are required.

Positive psychologists recognise that there is a need to identify characteristics and behaviours which are associated with optimal wellbeing [5, 10]. Nonetheless, research thus far has been limited to examining socio-demographic factors such as age, gender, ethnicity, and household income [8, 12]. It does however, seem plausible that prudent lifestyle behaviours, such as healthy eating, adequate sleep, physical activity, avoiding tobacco, and constraining alcohol consumption, may be associated with optimal wellbeing. Whilst lifestyle behaviours have been extensively examined in public health, research investigating associations between lifestyle behaviours and wellbeing have typically relied on single item measures of life satisfaction or happiness [13–15]. However, findings from previous studies show these single item measures only have small to moderate correlations with multi-dimensional measures of optimal wellbeing [5].

At an epidemiological level, an integrative approach to understanding associations between lifestyle behaviours and optimal wellbeing is needed. If associated, promoting lifestyle behaviours may provide an opportunity for increasing wellbeing, or vice versa. Identifying lifestyle behaviours that are associated with optimal wellbeing will provide a useful step in guiding future research and interventions aimed at promoting positive health. The purpose of this study is to integrate measures from public health and positive psychology to determine (1) the proportion of a large, demographically diverse sample of New Zealand adults meeting the criteria for optimal wellbeing and (2) associations between lifestyle behaviours and optimal wellbeing. This study will contribute to the limited research on lifestyle behaviours and multi-dimensional measures of optimal wellbeing.

## Methods

Data for this cross-sectional study were obtained from the Sovereign Wellbeing Index (Round 1); a survey on the health and wellbeing of a large, demographically diverse sample of New Zealand adults [16]. A web-based survey design was employed to collect data during September and October, 2012. Ethical approval to conduct the study was granted by the Auckland University of Technology Ethics Committee on 23 August, 2012 (AUTEC: 12/201).

The web-based survey design was chosen as it offered a number of advantages over traditional data collection modes (i.e. door-to-door or computer assisted telephone interviews). These advantages include the relative cost-effectiveness of the approach, the ability to overcome geographical constraints, and the minimisation of errors associated with data entry [17]. Recent reports indicate the proportion of New Zealand households with access to the internet (80 %) and landline telephones (85 %) is similar [18, 19].

### Participants

A commercial market research company (TNS Global, New Zealand office) was contracted to administer the web-based survey. Participants were recruited from the SmileCity database; the largest commercially available database in New Zealand. The database comprises 247,675 active members recruited through both offline (51 %) and online (49 %) sources [20].

The target sample size for the current study was 10,000 participants. The sample size was determined partly by financial constraints, and partly to obtain a reasonable precision of estimates. Eligible individuals included SmileCity database members aged over 18 years who had not participated in a survey within the last 7-days. There were no further exclusion criteria.

Email invites—with a link to the survey—were sent to 38,439 individuals randomly selected from the 229,032 eligible individuals. The survey was open to potential participants for 7-days. No follow-up invites were sent to individuals who did not complete the survey within the specified timeframe. All participants provided informed consent prior to entering the survey.

### Variables

The web-based survey included 134 questions on wellbeing, health and lifestyle, and socio-demographics. To enable international and national comparisons, the wellbeing component primarily comprised questions drawn from the European Social Survey (Round 6) [21] whilst the health and lifestyle component comprised questions primarily from the New Zealand Health Survey (2006) [22]. Measures specific to the current study only are discussed in detail below.

#### Optimal wellbeing

Optimal wellbeing was treated as a binary variable. The ten items (refer to Table 1) to measure optimal wellbeing were drawn from the European Social Survey (Round 6) [21]. A modified version of Huppert and So’s scale, reflecting changes made to two items between Rounds 3 and 6 of the European Social Survey, was used to calculate optimal wellbeing [5, 23, 24]. The two items which differed from the original scale were ‘*I love learning new things’* and ‘*There are people in my life who really care about me’.* These items were replaced with ‘*To what extent do you learn new things in your life’* and ‘*To what extent do you receive help and support from people you are close to when you need it’*, respectively [23, 24]. Hone et al. recently demonstrated moderate to strong agreement between the modified version of Huppert and So’s measure and other measures of optimal wellbeing [24].

**Table 1:** Constructs, features, items and thresholds used to calculate optimal wellbeing

Construct and features	Item (Likert scale; anchors)	Threshold
Positive emotion (required)		
• Happiness	Taking all things together, how happy would you say you are? *0–10; extremely unhappy-extremely happy*	≥ 8
Positive characteristics (4 of 5 required)		
• Emotional stability	In the past week, I felt calm and peaceful *1–4; none or almost none of the time-all or almost all of the time*	≥ 2
• Vitality	During the past week, you had a lot of energy? *1–4; none or almost none of the time-all or almost all*	≥ 3
• Optimism	I am always optimistic about my future *1–5; strongly disagree-strongly agree*	≥ 4
• Resilience	When things go wrong in my life it generally takes me a long time to get back to normal *1–5; strongly disagree-strongly agree; reverse score*	≥ 4
• Self-esteem	In general, I feel very positive about myself *1–5; strongly disagree-strongly agree*	≥ 4
Positive functioning (3 of 4 required)		
• Engagement	To what extent do you learn new things in your life? *0–6; not at all-a great deal*	≥ 5
• Competence	Most days I feel a sense of accomplishment from what I do *1–5; strongly disagree-strongly agree*	≥ 4
• Meaning	I generally feel that what I do in my life is valuable and worthwhile *1–5; strongly disagree-strongly agree*	≥ 4
• Positive relationships	To what extent do you receive help and support from people you are close to when you need it? *0–6; not at all-completely*	≥ 4

To be classified as meeting the criteria for optimal wellbeing individuals must (1) meet the threshold for positive emotion; (2) meet the threshold for four out of five features of positive characteristics; and (3) meet the threshold for three out of four features of positive functioning

The ten items used to measure optimal wellbeing combined both hedonic (feelings) and eudaimonic (functioning) aspects of wellbeing [5]. The items were rated on 4-point to 11-point Likert scales. All items were phrased in a positive direction except for the item measuring resilience, which was reverse coded. Optimal wellbeing was determined as meeting the thresholds for positive emotion (*happiness ≥ 8*); and four out of five features of positive characteristics (*vitality ≥ 3, optimism ≥ 4, resilience ≥ 4, self-esteem ≥ 4, emotional stability ≥ 2*); and three out of four features of positive functioning (*engagement ≥ 5, meaning ≥ 4, competence ≥ 4, positive relationships ≥ 4*) [5, 24]. Table 1 provides a summary of the constructs, features, items, and thresholds used to calculate optimal wellbeing.

#### Socio-demographic variables

Self-reported socio-demographic variables including gender, date of birth, ethnicity, and household income were collected as part of the web-based survey. In accordance with Statistics New Zealand’s Statistical Standard for Ethnicity, respondents were provided with the option of selecting multiple ethnic response categories [25]. Responses were coded into three independent categories (European/Other, Maori/Pacific, and Asian) using Statistics New Zealand Level 1 prioritised ethnic classifications [25]. Date of birth was used to calculate age with the survey start date as the reference. Continuous age was recoded into 10-yearly groupings according to Statistics New Zealand’s Statistical Standard for Age [26]. Finally, household income was stratified into tertiles to reflect low (≤ $40,000), moderate ($40,000-$90,000), and high (≥ $90,001) incomes.

#### Lifestyle behaviours

Ten lifestyle behaviours were included in the analysis including breakfast consumption, sugary drink consumption, fruit intake, vegetable intake, smoking, alcohol consumption, exercise, sedentary behaviour, sleep quality, and body mass index (BMI).

Questions to measure breakfast consumption, sugary drink consumption, fruit intake, vegetable intake, smoking, and alcohol consumption were drawn from the New Zealand Health Survey (2006), an annual door-to-door survey conducted by the Ministry of Health [22]. Respondents were asked to indicate how many days during the past week they had breakfast (*never, 1-2 days, 3-4 days, 5-6 days, 7 days*); how often during the past week they drank sugary beverages (*I don’t drink sugary drinks, less than once, 1-2 times, 3-4 times, 5-6 times, ≥7 times*); on average how many servings of fruit they had over the past week (*I don’t eat fruit, <1 serving/day, 1 serving/day; 2 servings/day; 3 servings/day, ≥ 4 servings/day*); and on average how many servings of vegetables they had over the past week (*I don’t eat vegetables, < 1 serving/day, 1 serving/day; 2 servings/day; 3 servings/day, ≥ 4 servings/day*) [22]. For smoking, respondents were asked if they smoke cigarettes regularly (*yes, no*) [22]. Alcohol consumption was assessed by asking respondents to indicate how often they have a drink containing alcohol *(I don’t drink alcohol, monthly or less, up to four times/month, up to three times/week, ≥ 4 times/week)* [22]*.*

Exercise was measured using a single item exercise frequency question which asked participants to report how often during the past week they exercised (*I didn’t exercise, 1-2 times, 3-4 times, 5-6 times, ≥ 7 times*) [14, 27]. Sedentary behaviour was measured using a single item sitting question [28]. Response options were adapted from their original format (*never, seldom, sometimes, often, always*) [28] to reflect the response scales used throughout the web-based survey (*none or almost none of the time, a little of the time, some of the time, most of the time, all or almost all of the time*).

Sleep quality was assessed using a question drawn from the European Social Survey (Round 6) Survey [29]. The question originates from the Center for Epidemiologic Studies Depression Scale [30] and has been used to measure restless sleep elsewhere [31, 32]. Respondents were asked to indicate how much of the time during the past week their sleep was restless (*none or almost none of the time, some of the time, most of the time, all or almost all of the time*).

Body mass index was derived using self-reported height and weight measures and was calculated as weight_kg_/(height_m_^2^). World Health Organization thresholds were used to categorise BMI as: underweight (≤18.4), normal weight (18.5–24.9), overweight (25.0–29.9), and obese (≥ 30.0) [33].

With the exception of BMI, all lifestyle variables are reported as per their original response scales.

### Data analysis

Optimal wellbeing was treated as the dependent variable. Participants’ data were, therefore, only included in the final analyses if a response was provided for each of the ten items used to calculate optimal wellbeing. Binary logistic regression analysis was used to determine associations between both demographic factors and lifestyle behaviours and optimal wellbeing (IBM SPSS Statistics version 19 for Windows). Crude, partially adjusted (adjusted for age, gender, ethnicity, and household income), and fully adjusted (adjusted for all socio-demographic and lifestyle variables concurrently) odds ratios were calculated. Bootstrapped 95 % confidence intervals (CI) were calculated using 1000 samples. The alpha was set at 0.05 to determine statistical significance. Missing data for lifestyle behaviours and socio-demographic variables were excluded pairwise.

## Results

### Participant characteristics

**Fig. 1:**
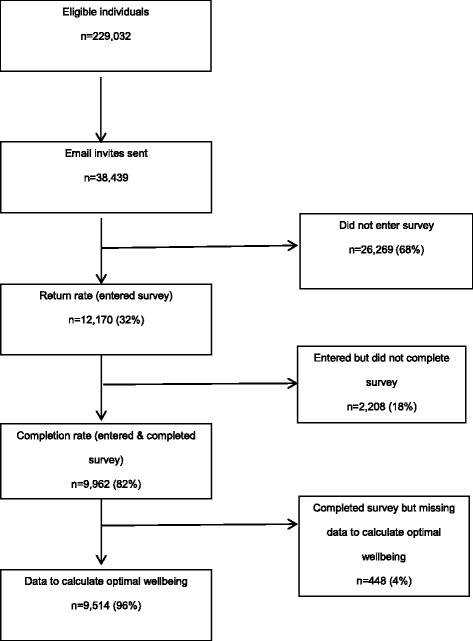
Flow diagram of participant recruitment

The return rate for the survey was 32 % (*n* = 12,170) and the completion rate was 82 % (*n* = 9962) (Fig. 1). Of those deemed to have completed the survey, data to calculate optimal wellbeing were available for 9514 (47 % male) participants. Sample characteristics for the current study are shown in Table 2.

**Table 2:** Sample characteristics and odds ratios for the relationship between lifestyle behaviours and optimal wellbeing (*n* = 9514)

	Total	Optimal wellbeing	Crude	Partially^3^ adjusted	Fully^4^ adjusted
	n (%)	n (%, 95 % CI)	OR^1^ (95 % CI^2^)	OR^1^ (95 % CI^2^)	OR^1^ (95 % CI^2^)
Gender					
Male	4478 (47)	1101 (25, 23–26)	1.00	1.00	1.00
Female	5013 (53)	1199 (24, 23–25)	0.96 (0.88–1.06)	1.10 (0.98–1.23)	1.02 (0.88–1.18)
Age, years					
< 20	221 (3)	41 (19, 13–24)	1.00	1.00	1.00
20–29	1856 (23)	359 (19, 18–21)	1.05 (0.75–1.56)	0.93 (0.60–1.59)	0.90 (0.55–1.50)
30–39	1472 (18)	305 (21, 19–23)	1.15 (0.82–1.75)	0.90 (0.57–1.53)	0.75 (0.46–1.27)
40–49	1413 (17)	303 (21, 19–24)	1.20 (0.85–1.82)	0.94 (0.59–1.62)	0.81 (0.50–1.42)
50–59	1326 (16)	336 (25, 23–28)	1.49 (1.06–2.21)*	1.23 (0.78–2.10)	0.98 (0.60–1.69)
60–69	1337 (16)	448 (34, 31–36)	2.21 (1.59–3.40)**	2.18 (1.40–3.69)**	1.43 (0.88–2.43)
70–79	495 (6)	198 (40, 36–44)	2.93 (2.03–4.39)**	3.36 (2.09–6.13)**	2.00 (1.16–3.51)*
≥ 80	54 (1)	17 (32, 19–44)	2.02 (0.94–3.92)*	1.91 (0.77–4.28)	1.19 (0.51–2.75)
Ethnicity					
European/Other	7093 (76)	1724 (24, 23–25)	1.00	1.00	1.00
Asian	1002 (11)	232 (23, 21–26)	0.94 (0.80–1.09)	1.26 (1.02–1.55)*	1.25 (0.97–1.59)
Maori/Pacific	1229 (13)	313 (26, 23–28)	1.06 (0.92–1.23)	1.20 (0.99–1.41)*	1.52 (1.23–1.89)**
Household income					
Low (≤ $40,000)	2366 (33)	481 (20, 19–22)	1.00	1.00	1.00
Mid ($40,001–$90,000)	2510 (36)	582 (23, 22–25)	1.18 (1.04–1.36)*	1.46 (1.27–1.67)**	1.30 (1.10–1.56)**
High (≥ $90,001)	2191 (31)	660 (30, 28–32)	1.69 (1.47–1.93)**	2.26 (1.93–2.61)**	1.84 (1.55–2.23)**
Restless sleep, how often past week					
None or almost none of the time	2182 (23)	754 (35, 33–37)	1.00	1.00	1.00
Sometimes	4397 (46)	1246 (28, 27–30)	0.75 (0.67–0.84)**	0.78 (0.68–0.89)**	0.83 (0.71–0.96)*
Most times	1915 (20)	217 (11, 10–13)	0.24 (0.20–0.29)**	0.27 (0.22–0.33)**	0.31 (0.24–0.39)**
All or almost all of the time	1009 (11)	84 (8, 7–10)	0.17 (0.13–0.22)**	0.20 (0.15–0.26)**	0.24 (0.17–0.32)**
Body mass index					
Normal weight	2660 (35)	645 (24, 23–26)	1.00	1.00	1.00
Underweight	148 (2)	28 (19, 13–25)	0.73 (0.46–1.08)	0.70 (0.39–1.14)	0.75 (0.37–1.30)
Overweight	2521 (33)	730 (29, 27–31)	1.27 (1.12–1.44)**	1.18 (1.01–1.38)*	1.24 (1.05–1.46)*
Obese	2375 (31)	491 (21, 19–22)	0.81 (0.71–0.93)**	0.75 (0.63–0.88)**	0.89 (0.74–1.07)
Alcohol, how often do you have a drink containing					
Up to 4 times/month	1707 (18)	450 (26, 24–28)	1.00	1.00	1.00
Never	2408 (26)	573 (24, 22–26)	0.87 (0.75–1.01)	0.83 (0.70–1.00)*	0.88 (0.71–1.08)
Monthly or less	2858 (31)	595 (21, 19–22)	0.73 (0.64–0.85)**	0.72 (0.61–0.85)**	0.76 (0.62–0.92)**
Up to 3 times/week	1273 (14)	342 (27, 24–29)	1.03 (0.86–1.22)	0.88 (0.71–1.06)	0.90 (0.72–1.11)
≥ 4 times/week	1088 (12)	300 (28, 25–30)	1.06 (0.89–1.27)	0.79 (0.65–0.98)*	0.87 (0.68–1.08)
Regular smoker					
Yes	1642 (17)	291 (18, 16–20)	1.00	1.00	1.00
No	7779 (83)	1997 (26, 25–27)	1.60 (1.40–1.85)**	1.44 (1.23–1.72)**	1.18 (0.98–1.45)
Exercise, how many times past week					
Don’t exercise	3379 (38)	614 (18, 17–19)	1.00	1.00	1.00
1–2 times/week	2552 (28)	612 (24, 22–26)	1.42 (1.24–1.62)**	1.46 (1.23–1.69)**	1.21 (1.02–1.43)*
3–4 times/week	1874 (21)	535 (29, 27–31)	1.80 (1.58–1.62)**	1.81 (1.52–2.13)**	1.39 (1.13–1.69)**
5–6 times/week	972 (11)	309 (32, 29–35)	2.10 (1.79–2.46)**	1.88 (1.56–2.29)**	1.37 (1.11–1.69)**
≥ 7 times/week	215 (2)	188 (37, 32–41)	2.59 (2.11–3.11)**	2.32 (1.78–3.10)**	1.61 (1.22–2.13)**
Sedentary levels, time spent sitting for the most part of each day past week					
All or almost all of the time	651 (7)	99 (15, 12–18)	1.00	1.00	1.00
None or almost none of the time	192 (2)	53 (28, 21–34)	2.13 (1.42–3.23)**	2.28 (1.38–3.72)**	1.87 (1.01–3.29)**
A little of the time	1263 (13)	363 (29, 26–31)	2.25 (1.77–2.96)**	1.91 (1.42–2.37)**	1.68 (1.20–2.49)**
Some of the time	4177 (44)	1169 (28, 27–29)	2.17 (1.72–2.78)**	1.88 (1.46–2.60)**	1.59 (1.18–2.26)**
Most of the time	3151 (33)	598 (19, 18–20)	1.31 (1.03–1.68)*	1.16 (0.91–1.62)	1.09 (0.79–1.57)
Vegetables, average servings per day over last week					
Don’t eat vegetables	107 (1)	11 (10, 5–16)	1.00	1.00	1.00
< 1 serving/day	859 (9)	115 (13, 11–16)	1.35 (0.77–2.99)	1.51 (0.68–7.07)	1.43 (0.58–7.14)
1 servings/day	2327 (25)	496 (21, 20–23)	2.36 (1.39–5.38)*	2.42 (1.13–11.89)*	1.75 (0.75–8.44)
2 servings/day	2432 (26)	577 (24, 22–25)	2.71 (1.58–6.09)**	3.01 (1.44–14.30)*	2.06 (0.86–10.17)
3 servings/day	2045 (22)	579 (28, 26–30)	3.45 (2.03–7.87)**	3.52 (1.62–16.79)**	2.21 (0.92–10.84)
≥ 4 servings/day	1563 (17)	502 (32, 30–34)	4.13 (2.42–9.27)**	4.22 (1.97–19.63)**	2.31 (0.97–11.54)
Fruit, average servings/day over last week					
Don’t eat fruit	323 (3)	42 (13, 9–17)	1.00	1.00	1.00
< 1 serving/day	1931 (21)	301 (16, 14–17)	1.24 (0.89–1.825)	1.07 (0.72–1.67)	0.88 (0.55–1.46)
1 servings/day	2826 (30)	661 (23, 22–25)	2.04 (1.50–2.89)**	1.68 (1.17–2.74)*	1.06 (0.66–1.78)
2 servings/day	2489 (27)	696 (28, 26–30)	2.60 (1.89–3.79)**	2.06 (1.43–3.27)**	1.13 (0.71–1.88)
3 servings/day	1134 (12)	356 (31, 29–34)	3.06 (2.21–4.54)**	2.38 (1.64–3.96)**	1.28 (0.78–2.19)
≥ 4 servings/day	642 (7)	226 (35, 32–39)	3.63 (2.60–5.49)**	2.59 (1.68–4.20)**	1.35 (0.80–2.35)
Breakfast, how many days over last week					
7 days/week	5255 (56)	1561 (30, 28–31)	1.00	1.00	1.00
Never	1060 (11)	153 (14, 12–17)	0.40 (0.33–0.47)**	0.50 (0.40–0.62)**	0.81 (0.62–1.08)
1–2 days/week	1116 (12)	178 (16, 14–18)	0.45 (0.37–0.53)**	0.54 (0.43–0.68)**	0.75 (0.58–0.93)*
3–4 days/week	943 (10)	196 (21, 18–23)	0.62 (0.52–0.73)**	0.80 (0.65–0.98)*	0.91 (0.71–1.17)
5–6 days/week	1020 (11)	200 (20, 17–22)	0.58 (0.49–0.67)**	0.60 (0.48–0.73)**	0.67 (0.52–0.84)**
Sugary drinks, how often over last week					
Don’t drink sugary drinks	2545 (27)	784 (31, 29–33)	1.00	1.00	1.00
< 1 time/week	1516 (16)	389 (26, 23–28)	0.78 (0.68–0.90)**	0.83 (0.69–0.99)*	0.86 (0.70–1.06)
1–2 times/week	2340 (25)	534 (23, 21–25)	0.66 (0.58–0.75)**	0.77 (0.66–0.90)**	0.82 (0.67–1.00)
3–4 times/week	1366 (15)	283 (21,19–23)	0.59 (0.51–0.68)**	0.72 (0.59–0.87)**	0.83 (0.66–1.03)
5–6 times/week	684 (7)	123 (18, 15–21)	0.49 (0.40–0.60)**	0.60 (0.46–0.78)**	0.73 (0.53–0.95)*
≥ 7 times/week	932 (10)	175 (19, 16–21)	0.52 (0.43–0.63)**	0.67 (0.54–0.85)**	0.92 (0.71–1.18)

*p < 0.05; **p < 0.01; ^1^odds ratio; ^2^bootstrapped 95 % confidence interval; ^3^adjusted for gender, age, ethnicity, income; ^4^adjusted for all demographic and lifestyle behaviours concurrently

The sample characteristics were compared to the estimated resident population in New Zealand during the June, 2012 to September, 2012 quarter [34]. Our sample was slightly over-represented by those in the lowest household income tertile (33 % vs. 32 %) and slightly under-represented by males (47 % vs. 49 %), and those aged 40–49 years (17 % vs. 19 %), 50–59 years (16 % vs. 18 %), and over 60 years (23 % vs. 26 %). Comparing the final sample characteristics to those that did not respond to the survey invite indicated non-respondents were over-represented by males (47 % vs. 51 %), those aged 18–29 years (26 % vs. 38 %), and those aged 30–39 years (18 % vs. 19 %).

### Optimal wellbeing

In total, 24 % (*n* = 3964) of the sample met the criteria for optimal wellbeing (Table 3). Over half the sample (54 %) met the criteria for positive functioning, whilst 41 % and 44 % met the criteria for positive emotions and positive characteristics, respectively (Table 3).

**Table 3:** Proportion of the sample meeting the criteria for optimal wellbeing (*n* = 9514)

Wellbeing features	n	%
Optimal wellbeing	2303	24
Positive emotion	3917	41
Positive characteristics (total meeting 4 of 5 features)	4219	44
Vitality	3712	39
Optimism	5918	62
Resilience	4357	46
Self-esteem	6431	68
Emotional stability	8414	88
Positive functioning (total meeting 3 of 4 features)	5103	54
Meaning	6801	72
Positive relationships	6488	68
Engagement	4210	44
Competence	5499	58

Optimal wellbeing was calculated as meeting thresholds for a) positive emotion and; b) four out of five features of positive characteristics – vitality, optimism, resilience, self-esteem, emotional stability and; c) three out of four features of positive functioning – meaning, positive relationships, engagement, competence [5, 24]

### Lifestyle behaviours and optimal wellbeing

Table 2 shows the crude, partially adjusted (age, gender, ethnicity, and household income), and fully adjusted (all demographic and lifestyle variables) odds ratios and bootstrapped 95 % CIs for optimal wellbeing and each of the socio-demographic and lifestyle variables assessed.

#### Socio-demographic variables

No associations between gender and optimal wellbeing were observed.

The trend across the three models indicates the likelihood of achieving the criteria for optimal wellbeing increases with age. The fully adjusted odds ratios show those aged 70–79 years were significantly more likely to report optimal levels of wellbeing compared to those aged less than 20 years (OR: 2.00, 95 % CI: 1.16–3.51, *p* < 0.05).

No association was observed between ethnicity and achieving the criteria for optimal wellbeing in the crude model. However, when adjusting for age, gender, and household income, Asian people and Maori/Pacific people were significantly more likely to meet the criteria for optimal levels of wellbeing compared to European/Other people. In the fully adjusted model the association between ethnicity and optimal wellbeing remained for Maori/Pacific people (1.52, 1.23–1.89, *p* < 0.01) but was negated for Asian people.

Household income was significantly associated with optimal wellbeing in each model. In the fully adjusted model those in the middle and highest income tertiles were 1.30 (1.10–1.56, *p* < 0.01) and 1.84 (1.55–2.23, *p* < 0.01) times more likely, respectively, to reach the criteria for optimal wellbeing compared to those in the lowest income tertile.

#### Nutrition variables

The average number of servings of fruit and vegetables consumed each day were positively and significantly associated with optimal wellbeing in the crude and partially adjusted models. However, these associations were negated in the fully adjusted model.

Sugary drink intake was inversely and significantly associated with optimal wellbeing in the crude and partially adjusted models. In the fully adjusted model consuming sugary drinks 5–6 times per week was associated with a decreased likelihood (0.73, 0.53–0.95, *p* < 0.05) of achieving the criteria for optimal wellbeing, compared to those who reported that they do not consume sugary drinks.

Compared to those that reported eating breakfast daily, eating breakfast 1-2 days per week (0.75, 0.58–0.93, *p* < 0.05) and 5-6 days per week (0.67, 0.52–0.84, *p* < 0.01) were associated with a decreased likelihood of being in the optimal wellbeing group.

The crude and partially adjusted models showed obese people were significantly less likely to have optimal levels of wellbeing compared to normal weight people, however, this association was negated in the fully adjusted model. Conversely, overweight people were significantly more likely to have optimal levels of wellbeing compared to normal weight people in all three models.

#### Health risk behaviours

Compared to smokers, being a non-smoker was associated with an increased likelihood of reaching the criteria for optimal wellbeing in the crude (1.60, 1.40–1.85, *p* < 0.01) and partially adjusted (1.44, 1.23–1.72, *p* < 0.01) models; however, these associations were negated in the fully adjusted model.

Compared to those that consume alcohol up to four times per month, drinking alcohol monthly or less was significantly associated with a decreased likelihood of being in the optimal wellbeing group in the crude (0.73, 0.64–0.85, *p* < 0.01), partially adjusted (0.72, 0.61–0.85, *p* < 0.01), and fully adjusted (0.76, 0.62–0.92, *p* < 0.01) models.

#### Exercise and sedentary behaviour

Exercise was positively and significantly associated with achieving the criteria for optimal wellbeing in all three models. Compared to those who reported doing no exercise, exercising seven or more times per week was associated with a 1.61 (1.22–2.13, *p* < 0.01) increased likelihood of being in the optimal wellbeing group in the fully adjusted model.

An inverse relationship between sedentary levels and optimal wellbeing was observed in all three models; decreases in sedentary behaviour were associated with an increased likelihood of meeting the criteria for optimal wellbeing. In the fully adjusted model, those who reported sitting none or almost none of the time during the past week were 1.87 (1.01–3.29, *p* < 0.01) times more likely to meet the criteria for optimal wellbeing.

#### Sleep

In all three models restless sleep was negatively associated with optimal wellbeing. In the fully adjusted model, having restless sleep all or almost all of the time was associated with a significantly decreased (0.24, 0.17–0.32, *p* < 0.01) likelihood of being in the optimal wellbeing group compared to those reporting restless sleep none or almost none of the time.

## Discussion

To develop positive health and wellbeing interventions a better understanding of the characteristics and behaviours associated with optimal wellbeing is needed. In the present study a multi-dimensional measure of optimal wellbeing was used to classify those with the highest levels of wellbeing in a large and diverse sample of New Zealand adults. Our findings show 24 % of the sample met the criteria for optimal wellbeing. The second aim of the study was to integrate measures from public health and positive psychology to determine lifestyle behaviours associated with a multi-dimensional measure of optimal wellbeing. In the fully adjusted model, optimal wellbeing was positively associated with exercise, inversely associated with sedentary behaviour, and negatively associated with sleep.

In previous research on life satisfaction, New Zealand ranks similar or just below the highest ranked Scandinavian nations [35]. Comparing our findings to the largest study of optimal wellbeing in Europe suggests that proportion of New Zealanders meeting the criteria for optimal wellbeing in the current sample is comparable to Sweden (24 %; ranked 7 of 22) [5]. Nevertheless, the 24 % reported in our sample is substantially lower than the prevalence of optimal wellbeing in the highest ranked country, Denmark (41 %) [5]. Our data indicates that individuals in our sample who were more likely to achieve the criteria for optimal wellbeing were those with higher household incomes and those aged 70–79 years. It was also interesting to observe, that in contrast to previous research reporting lower levels of satisfaction with life among Maori and Pacific people [36, 37], we found no evidence to support this relationship. In contrast, we found those who identified as Maori or Pacific were more likely to achieve optimal levels of wellbeing compared to those who identified as European. Whilst these ethnic differences in optimal wellbeing warrant further investigation, the discrepancy between our research and others may in part be explained by the wellbeing measures used. We used a broader criteria to measure optimal wellbeing which takes into account dimensions such as positive relationships and meaning and purpose. Maori and Pacific people may have scored higher on these dimensions due to the cultural value placed on philosophies such as *whanau ora* and *hauora* [38]. For example, whanau ora emphasises the family and community whilst hauora emphasises physical, mental and emotional, social, and spiritual wellbeing approaches to health [38].

In the present study, we extend previous research on life satisfaction [13] and happiness [14] to show for the first time that sleep, exercise, and sedentary behaviour are independently associated with a multi-dimensional measure of optimal wellbeing. Although exercise, and to a lesser extent minimising sedentary behaviour, are important public health priorities, sleep is often overlooked. There is now increasing evidence to show that sleep is associated with health outcomes [39]. Our findings also indicate that reporting restless sleep almost all of the time was associated with 0.24-fold decreased likelihood of meeting the criteria for optimal wellbeing, compared with those reporting restless sleep almost none of the time. This is concerning as there is accumulating evidence to suggest that there has been a global reduction in sleep [40, 41]. Given the potential implications for health and wellbeing increased efforts should be made to raise awareness of strategies to improve sleep quality.

To a lesser degree, associations between optimal wellbeing and breakfast consumption, sugary drink intake, BMI, and alcohol consumption were also observed in the current study. Similar associations between breakfast consumption and happiness have been observed previously [14]. In our study we found that compared to those who drink alcohol up to four times per month, drinking alcohol monthly or less was associated with a 0.76-fold decreased likelihood of achieving the criteria for optimal wellbeing. Though not significant Piqueras et al. [14] reported a similar trend; compared to those that never drink those who reported drinking were more likely to be classified as happy (OR 1.07; *p* = 0.52). One possible explanation for this somewhat unexpected finding is that those occasionally drinking may be doing so in social environments thereby benefiting from social interaction and enhanced positive relationships. Finally, consuming sugary drinks 5-6 times per week was associated with a 0.73-fold decreased likelihood of achieving the criteria for optimal wellbeing. Limiting sugar is, therefore, likely to have implications from both a health and wellbeing perspective.

The relationship between lifestyle behaviours and optimal wellbeing is complex and likely to be bidirectional. On the one hand, if individuals feel optimistic, energetic, confident, and supported they are probably more likely to engage in positive lifestyle behaviours [42]. Alternatively, there is also evidence to support the claim that optimal wellbeing is enhanced by healthy lifestyle behaviours [43]. Specifically, experimental research in human and animal studies shows that engaging in healthy behaviours such as exercise [44], healthy eating [45, 46], and quality sleep [47] reduces inflammation and enhances BDNF. Reducing inflammation and enhancing BNDF expression promotes neuroplasticity which is important for dimensions of optimal wellbeing related to creativity, exploration, and curiosity [43].

It is pertinent to note that three quarters of the sample in the current study were not meeting the criteria for optimal wellbeing. It is therefore evident that further efforts need to be made promote and increase optimal wellbeing. Whilst the causal relationship between lifestyle behaviours and optimal wellbeing cannot be determined from our data, existing evidence supports the claim that the relationship is likely to be bidirectional. Our findings, together with the literature, provide support for holistic interventions which integrate the promotion of lifestyle behaviours and the dimensions underpinning optimal wellbeing (e.g. relationships, self-esteem, and resilience). Our research shows the lifestyle variables which should be targeted in such interventions include sleep, exercise, and sedentary behaviour, and to a lesser degree sugary drink consumption and breakfast intake.

### Limitations

The findings from this study should be considered in light of the limitations. Firstly, although the web-based survey design offered a number of advantages, the response rate was low (32 %). Comparisons to the estimated resident population during the September 2012 quarter indicate our sample was slightly over-represented by younger adults and slightly under-represented by those aged over 60 years. Secondly, the current study relied on self-reported lifestyle behaviours, rather than observational measures. There is therefore a possibility that individuals were subject to social desirability bias. Finally, the cross-sectional nature of the data precludes the ability to infer causation. The relationship between lifestyle behaviours and optimal wellbeing is complex and likely to be bidirectional. Intervention and mechanistic studies are required to further progress our understanding of this relationship. The findings from this study provide a starting point for determining the most pertinent lifestyle variables to include in such research.

## Conclusion

In this study, two research fields—positive psychology and public health—have been integrated to examine the relationship between lifestyle behaviours and optimal wellbeing. The current study contributes to the limited wellbeing research in New Zealand to show almost a quarter of a large and demographically diverse sample of New Zealanders are meeting the criteria for optimal wellbeing. This study also extends current international knowledge to show sleep, exercise, and sedentary behaviour, and to a lesser degree breakfast consumption, sugary drink intake, BMI, and alcohol consumption are associated with a broader, more complex notion of wellbeing. It is likely this relationship between lifestyle behaviours and optimal wellbeing is bidirectional giving rise to the debate that holistic approaches are needed to promote positive health.

## Abbreviations

BDNF: Brain derived neurotophic factor
BMI: Body mass index
CI: Confidence interval
OR: Odds ratio.

## References

[1] World Health Organization (2006). Constitution of the World Health Organization. Basic Documents.

[2] Ryff CD, Singer B (1998). The contours of positive human health. Psychol Inq.

[3] Ryff CD, Singer B, Love GD (2004). Positive health: connecting well–being with biology. Philos T Roy Soc B.

[4] Seligman M (2008). Positive health. Appl Psych Meas.

[5] Huppert F, So TC (2013). Flourishing across Europe: application of a new conceptual framework for defining well-being. Soc Indic Res.

[6] Seligman M, Csikszentmihalyi M (2000). Positive psychology: an introduction. Am Psychol.

[7] Ryff CD, Keyes CLM (1995). The structure of psychological well-being revisited. J Pers Soc Psychol.

[8] Keyes CLM (2002). The mental health continuum: from languishing to flourishing in life. J Health Soc Behav.

[9] Huppert FA, Marks N, Clark A, Siegrist J, Stutzer A, Vitterso J (2009). Measuring well-being across Europe: description of the ESS well-being module and preliminary findings. Soc Indic Res.

[10] Diener E, Wirtz D, Tov W, Kim-Prieto C, Choi D-w, Oishi S (2010). New well-being measures: short scales to assess flourishing and positive and negative feelings. Soc Indic Res.

[11] Seligman M (2012). Flourish: A visionary new understanding of happiness and well-being.

[12] Schotanus-Dijkstra M, Pieterse ME, Drossaert CHC, Westerhof GJ, de Graaf R, ten Have M, et al. What factors are associated with flourishing? Results from a large representative national sample. J Happiness Stud. 2015. doi:10.1007/s10902-015-9647-3.

[13] Grant N, Wardle J, Steptoe A (2009). The relationship between life satisfaction and health behavior: a cross-cultural analysis of young adults. Int J Behav Med.

[14] Piqueras JA, Kuhne W, Vera-Villarroel P, Van Straten A, Cuijpers P: Happiness and health behaviours in Chilean college students: a cross-sectional survey. BMC Public Health. 2011;11. doi:10.1186/1471-2458-1111-1443. 10.1186/1471-2458-11-443 PMC3125376 21649907

[15] Ministry of Health (2012). The health of New Zealand adults 2011/12: key findings of the New Zealand health survey.

[16] Jarden A, Mackay L, White K, Schofield G, Williden M, Hone L (2013). The Sovereign New Zealand Wellbeing Index. Psychology Aotearoa.

[17] van Gelder MM, Bretveld RW, Roeleveld N (2010). Web-based questionnaires: the future in epidemiology?. Am J Epidemiol.

[18] Statistics New Zealand (2013). Household use of information and communication technology: 2012.

[19] Census QuickStats about transport and communications. 2013. [http://www.stats.govt.nz/Census/2013-census/profile-and-summary-reports/quickstats-transport-comms.aspx]. Accessed 18 November 2015.

[20] Ltd SC (2012). ESOMAR: 27 Questions.

[21] European Social Survey (2012). ESS Round 6 source questionnaire.

[22] Ministry of Health (2006). 2006/07 New Zealand health survey: adult questionnaire.

[23] European Social Survey (2013). Round 6 module on personal and social wellbeing: final module in template.

[24] Hone L, Jarden A, Schofield G (2014). Measuring flourishing: the impact of operational definitions on the prevalence of high levels of wellbeing. IJW.

[25] Statistical standard for ethnicity. [http://www.stats.govt.nz/surveys_and_methods/methods/classifications-and-standards/classification-related-stats-standards/ethnicity.aspx]. Accessed 08 December 2012.

[26] Statistical standard for age. [http://www.stats.govt.nz/surveys_and_methods/methods/classifications-andstandards/classification-related-stats-standards/age.aspx]. Accessed 09 December 2015.

[27] Sullivan C, Oakden J, Young J, Butcher H, Lawson R (2003). Obstacles to action: a study of New Zealanders’ physical activity and nutrition, Technical report.

[28] Baecke JAH, Burema J, Frijters ER (1982). A short questionnaire for the measurement of habitual physical activity in epidemiological studies. Am J Clin Nutr.

[29] European Social Survey (2006). ESS Round 3 source questionnaire.

[30] Radloff LS (1977). The CES-D Scale: a self-report depression scale for research in the general population. Appl Psychol Meas.

[31] Burgard SA, Ailshire JA (2009). Putting work to bed: stressful experiences on the job and sleep quality. J Health Soc Behav.

[32] Kutner NG, Bliwise DL, Zhang R (2004). Linking race and well-being within a biopsychosocial framework: variation in subjective sleep quality in two racially diverse older adult samples. J Health Soc Behav.

[33] Body mass index: BMI. [www.euro.who.int/en/health-topics/disease-prevention/nutrition/a-healthy-lifestyle/body-mass-index-bmi]. Accessed 09 December 2015.

[34] Population estimates. [www.stats.govt.nz/infoshare/SelectVariables.aspx?pxID=598e3be1-1c95-494c-ba1a-af1101e65492]. Accessed 09 December 2015.

[35] Diener E, Diener M (1995). Cross-cultural correlates of life satisfaction and self-esteem. J Pers Soc Psychol.

[36] Croft AGW, Lawson R. Applying the international wellbeing index to investigate subjective wellbeing of New Zealanders with European and with Maori heritage. Kotuitui: New Zealand Journal of Social Sciences Online. 2008;3(1):57-72.

[37] Sibley C, Harré N, Hoverd W, Houkamau C (2011). The gap in the subjective wellbeing of Māori and New Zealand Europeans widened between 2005 and 2009. Soc Indic Res.

[38] Chant L (2011). Whanau ora: Hauora Maori models for kotahitanga/co-operative co-existence with non-Maori. AlterNative: An Int J Indigenous Peoples.

[39] Cappuccio FP, D’Elia L, Strazzullo P, Miller MA (2010). Sleep duration and all-cause mortality: a systematic review and meta-analysis of prospective studies. Sleep.

[40] Alvarez GG, Ayas NT (2004). The impact of daily sleep duration on health: a review of the literature. Prog Cardiovasc Nurs.

[41] Chaput J-P, Tremblay A (2012). Insufficient sleep as a contributor to weight gain: an update. Current Obesity Reports.

[42] Salovey P, Detweiler JB, Steward WT, Rothman AJ (2000). Emotional states and physical health. Am Psychol.

[43] Gomez-Pinilla F (2008). The influences of diet and exercise on mental health through hormesis. Ageing Res Rev.

[44] Ferris LT, Williams JS, Shen CL (2007). The effect of acute exercise on serum brain-derived neurotrophic factor levels and cognitive function. Med Sci Sports Exerc.

[45] Sharma S, Fulton S (2013). Diet-induced obesity promotes depressive-like behaviour that is associated with neural adaptations in brain reward circuitry. Int J Obes.

[46] Kim JH, Kim SJ, Lee WY, Cheon YH, Lee SS, Ju A (2013). The effects of alcohol abstinence on BDNF, ghrelin, and leptin secretions in alcohol-dependent patients with glucose intolerance. Alcohol Clin Exp Res.

[47] Sei H, Saitoh D, Yamamoto K, Morita K, Morita Y (2000). Differential effect of short-term REM sleep deprivation on NGF and BDNF protein levels in the rat brain. Brain Res.

